# “I am in favour of organ donation, but I feel you should opt-in”—qualitative analysis of the #options 2020 survey free-text responses from NHS staff toward opt-out organ donation legislation in England

**DOI:** 10.1186/s12910-024-01048-6

**Published:** 2024-04-20

**Authors:** Natalie L. Clark, Dorothy Coe, Natasha Newell, Mark N. A. Jones, Matthew Robb, David Reaich, Caroline Wroe

**Affiliations:** 1 South Tees Hospitals NHS Foundation Trust, Middlesbrough, North Yorkshire, England UK; 2 Newcastle-Upon-Tyne Hospitals NHS Foundation Trust, Newcastle Upon Tyne, Tyne and Wear, England UK; 3 Centre for Process Innovation, Sedgefield, County Durham, England UK; 4 NHS Blood and Transplant, Bristol, England UK

**Keywords:** Organ donation, Legislation, National Health Service, Qualitative

## Abstract

**Background:**

In May 2020, England moved to an opt-out organ donation system, meaning adults are presumed to be an organ donor unless within an excluded group or have opted-out. This change aims to improve organ donation rates following brain or circulatory death. Healthcare staff in the UK are supportive of organ donation, however, both healthcare staff and the public have raised concerns and ethical issues regarding the change. The #options survey was completed by NHS organisations with the aim of understanding awareness and support of the change. This paper analyses the free-text responses from the survey.

**Methods:**

The #options survey was registered as a National Institute of Health Research (NIHR) portfolio trial [IRAS 275992] 14 February 2020, and was completed between July and December 2020 across NHS organisations in the North-East and North Cumbria, and North Thames. The survey contained 16 questions of which three were free-text, covering reasons against, additional information required and family discussions. The responses to these questions were thematically analysed.

**Results:**

The #options survey received 5789 responses from NHS staff with 1404 individuals leaving 1657 free-text responses for analysis. The family discussion question elicited the largest number of responses (66%), followed by those against the legislation (19%), and those requiring more information (15%). Analysis revealed six main themes with 22 sub-themes.

**Conclusions:**

The overall #options survey indicated NHS staff are supportive of the legislative change. Analysis of the free-text responses indicates that the views of the NHS staff who are against the change reflect the reasons, misconceptions, and misunderstandings of the public. Additional concerns included the rationale for the change, informed decision making, easy access to information and information regarding organ donation processes. Educational materials and interventions need to be developed for NHS staff to address the concepts of autonomy and consent, organ donation processes, and promote family conversations. Wider public awareness campaigns should continue to promote the positives and refute the negatives thus reducing misconceptions and misunderstandings.

**Trial registration:**

National Institute of Health Research (NIHR) [IRAS 275992].

**Supplementary Information:**

The online version contains supplementary material available at 10.1186/s12910-024-01048-6.

## Background

In England May 2020, Max and Kiera’s Law, also known as the Organ Donation (Deemed Consent) Bill, came into effect [1, 2]. This means adults in England are now presumed to have agreed to deceased organ donation unless they are within an excluded group, have actively recorded their decision to opt-out of organ donation on the organ donor register (ODR), or nominated an individual to make the decision on their behalf [1, 2]. The rationale for the legislative change is to improve the organ donation rates and reduce the shortage of organs available to donate following brain or circulatory death within the UK [2–4]. This is particularly important considering the growing number of patients awaiting a transplant. Almost 7000 patients were waiting in the UK at the end of March 2023 [5]. Wales was the first to make the legislative change in December 2015, followed by Scotland in March 2021 and lastly Northern Ireland in June 2023 [2]. Following the change in Wales, consent rates had increased from 58% in 2015/16 to 77% in 2018/19 [6], suggesting the opt-out system can significantly increase consent, though it further suggests that it might take a few years to fully appreciate the impact [7, 8]. Spain, for example, has had an opt-out legislation since 1979 with increases in organ donation seen 10 years later [9].

Research, however, has raised concerns from both the public and healthcare staff regarding the move to an opt-out system. These concerns predominantly relate to a loss of freedom and individual choice [9, 10], as well as an increased perception of state ownership of organs [10–12] after death. Healthcare staff additionally fear of a loss of trust and a damaged relationship with their patients [9, 11]. These concerns are frequently linked to emotional and attitudinal barriers towards organ donation, understanding and acceptance [9]. Four often referenced barriers include (1) jinx factor: superstitious beliefs [13–15]; (2) ick factor: feelings of disgust related to donating [13–15]; (3) bodily integrity: body must remain intact [13–15]; (4) medical mistrust: believing doctors will not save the life of someone on the ODR [13–15]. The latter barrier is mostly reported by the general public in countries with opt-out systems [13, 14, 16] although medical mistrust does feature as a barrier across all organ donation systems. In addition, it is a reported barrier healthcare staff believe will occur in the UK under an opt-out system [9, 16].

Deceased donation from ethnic minority groups is low in the UK, with family consent being a predominant barrier in these groups. Consent rates are 35% for ethnic minority eligible donors compared to 65% for white eligible donors [5]. The reasons for declining commonly relate to being uncertain of the person’s wishes and believing it was against their religious/cultural beliefs. Healthcare staff, particularly in the intensive care setting, have expressed a lack of confidence in communication and supporting ethnic minority groups because of language barriers and differing religious/cultural beliefs to their own [17]. However, one study has highlighted that generally all religious groups are in favour of organ donation with respect to certain rules and processes. Therefore, increasing knowledge amongst healthcare staff of differing religious beliefs would improve communication and help to sensitively support families during this difficult time [18, 19]. However, individually and combined, the attitudinal barriers, concerns towards an opt-out system, and lack of understanding about ethnic minority groups, can have a significant impact within a soft opt-out system whereby the family are still approached about donation and can veto if they wish [11, 12, 20].

The #options survey [21] was completed online by healthcare staff from National Health Service (NHS) organisations in North-East and North Cumbria (NENC) and North Thames. The aim was to gain an understanding of the awareness and support to the change in legislation. The findings of the survey suggested that NHS staff are more aware, supportive, and proactive about organ donation than the general public, including NHS staff from religious and ethnic minority groups. However, there were still a number who express direct opposition to the change in legislation due to personal choice, views surrounding autonomy, misconceptions or lack of information. This paper will focus on the qualitative analysis of free-text responses to three questions included in the #options survey. It aims to explore the reasons for being against the legislation, what additional information they require to make a decision, and why had they not discussed their organ donation decision with their family. It will further explore a subset analysis of place of work, ethnicity, and misconceptions. The findings will aid suggestions for future educational and engagement work.

## Methods

### Design, sample and setting

The #options survey was approved as a clinical research study through the integrated research application system (IRAS) and registered as a National Institute of Health Research (NIHR) portfolio trial [IRAS 275992]. The survey was based on a previously used public survey [22] and peer reviewed by NHS Blood and Transplant (NHSBT). The free-text responses used in #options were an addition to the closed questions used in both the #options and the public survey. Due to the COVID-19 pandemic, the start of the survey was delayed by 4 months, opening for responses between July to December 2020. All NHS organisations in the NENC and North Thames were invited to take part. Those that accepted invitations were supplied with a communication package to distribute to their staff. All respondents voluntarily confirmed their agreement to participate in the survey at the beginning. The COnsolidated criteria for REporting Qualitative research (COREQ) checklist was used to guide analysis and reporting of findings [23], see Supplementary material 1.

### Data collection and analysis

The survey contained 16 questions, including a brief description of the change in legislation. The questions consisted of demographic details (age, sex, ethnicity, religion), place of work, and if the respondent had contact with or worked in an area offering support to donors and recipients. Three of the questions filtered to a free-text response, see Supplementary material 2. These responses were transferred to Microsoft Excel to be cleaned and thematically analysed by DC. Thematic analysis was chosen to facilitate identification of groups and patterns within large datasets [24]. Each response was read multiple times to promote familiarity and initially coded. Following coding, they were reviewed to allow areas of interest to form and derive themes and sub-themes. Additional subsets were identified and analysed to better reflect and contrast views. This included, at the request of NHSBT, the theme of ‘misconceptions’. The themes were reviewed within the team (DC, CW, NK, NC, MJ) and shared with NHSBT. Any disagreements were discussed and agreed within the team.

## Results

Overall, the #options survey received 5789 responses from NHS staff. The COVID-19 pandemic further impacted on NHS organisations from North Thames to participate, resulting in respondents predominantly being from NENC (86%). Of the respondents, 1404 individuals (24%) left 1657 free-text responses for analysis. The family discussion question elicited the largest number of responses, accounting for 66% of the responses (*n* = 1088), followed by against the legislation at 19% (*n* = 316) and more information needed at 15% (*n* = 253). The responses to the against legislation question provided the richest data as they contained the most information. Across the three questions, there were six main themes and 22 sub-themes, see Table 1. The large number of free-text responses illustrate the multifaceted nature of individuals views with many quotes containing overlap between themes and sub-themes.

**Table 1:** Themes and sub-themes per question

Question	Theme	Sub-theme	Example [ID]
**I am against the legislation – Can you help us understand why you are against this legislation?**	Loss of autonomy	Informed consent	*I do not believe that consent can be said to have been obtained just because someone hasn’t recorded their wish to opt-out. [R1908]*
		Access	*Personally I think it was easier when people carried organ donation cards and was a definite visual and personal choice. Not everyone has the access to the internet or is able to cope with technology in order to opt-out… [R958]*
		Lack of awareness	*I was unaware of these changes and I do not wish to donate my organs when I die. Not a lot of people will know about these changes and our choices will be taken away from us when we die. [R868]*
		State ownership	*I don’t believe the State should have default rights over a person’s organs upon their death. [R2055]*
	Consequences	Mistakes	*There may be people who forget to opt-out and their wishes then not carried out. [R2121]*
		Loss of trust	*Don’t trust doctors in regards to organ donation. [R3010]*
		Family distress	*If the person did not decide to be an organ donor nor not to be, this will leave a huge burden on next of kin/family to make that decision. [R1652]*
	Legislation	Evidence-base & rationale	*Research shows that this measure doesn’t significantly improve “donation” rates. [R2493]*
		Organ choice	*I feel you should have the right to pick what you want to donate. [R3936]*
	Religion and culture	Bodily integrity	*I would like my body to be treated with dignity and not to have any organs removed. [R1839]*
		Brain death	*As a practicing Roman Catholic, it's wrong to take a person's life. Although a patient might be on life support with no hope of recovery, they are still alive at the point of organ retrieval. "Brain dead" is diagnosed even though the heart is still beating, therefore the patient is still alive. [R45]*
		Against	*Against my religion. [R5185]*
**I need more information to decide – What information would you like to help you decide?**	“Everything”	Family influence	*How relatives are informed and how they can opt-out of it on behalf of their loved ones. [R459]*
		Process(es) of donation	*What is the process for having organs taken following death? Who needs to be consulted and what’s the procedure? [R200]*
		Publicity	*What has been communicated to the general public, and to our patients? As it seems totally lacking. People can't make an informed decision if they weren't informed. [R5377]*
		Systems	*Where are my details stored if I opt out and can mistakes be made in relation to my choices? [R1287]*
		Evidence-base	*Further understanding of the issue and the background to the change. [R2524]*
**Have you discussed your decision with a family member? If no, can you help us understand what has stopped you discussing this with your family?**	Priority and relevance	Autonomy	*I decided to be an organ donor as it is my decision and didn’t feel the need to discuss it. [R482]*
		Too difficult	*It has just never come up in conversation and no-one likes to talk about death do they. [R3788]*
		Agreement	*The topic has not arisen but I am confident there would be no objections from my family. [R1898]*
		No family	*I'm a single parent and my children are too young to discuss this with. [R93]*
		No decision	*The topic hasn't come up in conversation and I am still undecided as to what I will do. [R1623]*

### Respondent characteristics

In comparison to the whole #options survey respondents, the free-text response group contained proportionally more males (21% vs 27%), less females (78% vs 72%), and marginally more 18–24year-olds (7% vs 8%), respectively. There were 5% more 55 + year olds in the free-text group, however all other age groups were between 2–3% lower when compared to the whole group. Additionally, the free-text group were more ethnically diverse than the whole group (6.9% vs 15.4%), with all named religions also having a higher representation (3.9% vs 7.3%), respectively.

### Question one: I am against the legislation – Can you help us understand why you are against this legislation?

Of the three questions, this elicited the largest number of responses from males (*n* = 94, 30%), those aged over 55 years (*n* = 103, 33%), and ethnic minority responders (*n* = 79, 25%). Subset analysis of place of employment indicates 27% were from the transplant centre (*n* = 84), 8% were from the mental health trust (*n* = 26), and 4% from the ambulance trust (*n* = 14). Thematic analysis uncovered four main themes and 12 sub-themes from the responses, with the predominant theme being a perceived loss of autonomy.

#### Theme one: loss of autonomy

Respondents’ reasons for a loss of autonomy were categorised into four sub-themes. Firstly, calling into question the nature of informed consent and secondly, peoples’ awareness of the legislative change. One respondent stated individuals need to be *“fully aware and informed” [R2943]* in order to have consented to organ donation. However, one respondent stated that they believe individuals have *“not [been] informed well” [R930]* and thus *“if people are not aware of it, how are they making a choice on what happens to their organs” [R1166]*. It was suggested that awareness of the change may have *“been overshadowed by COVID” [R4119]*.

Furthermore, there was concerns regarding the means to record an opt-out decision, specifically to those that are *“not tech savvy” [R167]*, *“homeless” [R5721]*, *“vulnerable” [R4553]*, and *“elderly” [R2155]*. Therefore, removing that individual’s right to record their decision due to being at a disadvantage.

Finally, respondents expressed concerns of a move to an authoritarian model of State ownership of organs. This elicited strong, negative reactions from individuals under the belief the State would own and *“harvest”* a person’s organs under a deemed consent approach, with some removing themselves as a donor consequently, *“I am furious that the Government has decided that my organs are theirs to assign. It is MY gift to give, not theirs. I have now removed myself as a long-standing organ donor.” [R593]*.

#### Theme two: consequences

Following respondents stating their reason for being against the legislative change, they discussed further what they believed to be the consequences of an opt-out legislation, with a focus on trust. Respondents cited a lack of trust towards the system, *“I have no Trust in the UK government” [R5374]*, with some surprisingly citing a lack of trust towards healthcare professionals, *“Don’t trust doctors in regard to organ donation” [R3010],* as well as a fear of eroding trust with the general public, *“This brings the NHS Organ Donation directly into dispute with the public.” [R1237].* Respondents additionally believed the legislative change would lead to an increase in mistakes i.e., organ’s being removed against a person’s wishes by presuming, *“not convinced that errors won't be made in my notifying my objection and that this won't be dealt with or handed over correctly” [R3018].* Finally, it is believed this change would also lead to, *“additional upset” [R587],* for already grieving families.

#### Theme three: legislation

Respondents were additionally against the legislation itself as they believed it lacked an evidence-base to prove it is successful at increasing the numbers of organs donated. As well as this, respondents perceived the legislation as one that removed the donor’s choice as to which organs they want to donate, some with a religious attribute *“I don't mind donating but would like choice of what I like to i.e., not my cornea as for after life I want to see where I am going.” [R5274].*

#### Theme four: religion and culture

Religion and culture was another common theme with sub-themes relating to maintaining bodily integrity following death and the lack of clarity around the definition of brain death. Many others stated that organ donation is against their religion or, were *“unsure whether organ donation is permissible” [R1067].*

### Question two: I need more information to decide—What information would you like to help you decide?

This question elicited the most responses from females (*n* = 188, 74%), those aged over 55 years (*n* = 80, 32%), with 19% being from ethnic minority groups (*n* = 49). Subset analysis of place of employment indicates 18% were from the transplant centre (*n* = 46), 8% were from the mental health trust (*n* = 18), and 9% from the ambulance trust (*n* = 23). Thematic analysis uncovered a main theme of *“everything”*. There were many responses that did not specify what information was required, but indicated that more general information on organ donation was required, within this there were five sub-themes.

#### Sub-themes:

The first sub-theme identified a request for information around the influence a family has on the decision to donate and the information that will be provided to families. This included providing *“emotional wellbeing” [R162]* support, and information on whether families can *“appeal against the decision” [R539]* or *“be consulted” [R923]* following their loved one’s death. This was mainly requested by those employed by transplant centres.

The second request was for information on the *“process involved after death for organ retrieval” [R171]*, predominantly by ethnic minority groups and those employed by the mental health trusts, with specific requests on confirming eligibility. Other examples of requested information on the process and pathway included *“how the organs will be used” [R1086]*, *“what will be donated” [R1629]*, and *“who benefits from them” [R3730]*.

The third request was information regarding the publicity strategy to raise awareness of the legislative change. Many of the respondents stated they did not think there was enough *“coverage in the media” [R3668].* Additional considerations of public dissemination were to ensure it was an “*easy read update” [R137*3*]*, specifically for *“the elderly or those with poor understanding of English who may struggled with the process” [R1676]*.

The fourth request was information around the systems in place to record a decision. There were additional requests for the opt-out processes if someone was within the excluded group and *“what safeguards are in place” [R3777],* as well as what if individuals change their mind and the ease of recording this new decision.

Finally, and similarly to the first question, the fifth request was information for an evidence-base. Respondents stated that they, *“would like to know the reasons behind this change” [R3965]*, believing that if they had a greater understanding then this might increase their support towards the legislative change.

### Question three: Have you discussed your decision with a family member? If no, can you help us understand what has stopped you discussing this with your family?

The free-text responses to analyse were from those who responded “No” to, “Have you discussed your decision with a family member?”. This received 1430 responses with females (*n* = 1025, 27%) predominantly answering “No”. However, not everyone left a free-text response, leaving 1088 comments for analysis. These were predominantly made by those aged over 55 years (*n* = 268, 24%), with 5% being from ethnic minority groups (*n* = 49). Subset analysis of the 1088 responses regarding place of employment indicated 14% were from the transplant centre (*n* = 147), 7% were from the mental health trust (*n* = 78), and 9% from the ambulance trust (*n* = 96). The analysis uncovered a main theme of priority and relevance made up of five sub-themes.

#### Sub-themes:

The first sub-theme identified one reason to be that it was their *“individual decision” [R3]* and there would be *“nothing to be gained” [R248]* from a discussion. Some respondents stated that despite a lack of discussion, their family members would assume their wishes in relation to organ donation and support these, *“I imagine they are all of the same mindset” [R4470].* However, some stated their reasons to be because they *“don’t have a family” [R1127]* to discuss this with or have *“young ones whose understanding is limited about organ donation” [R356]*. Positively, there were several respondents who suggested the question had acted as a prompt to speak to their family.

Another reason stated by respondents was that they found the topic to be too difficult to discuss due to *“recent bereavements” [R444], “current environment” [R441]*, and *“a reluctance to address death” [R4486]*. As evident in the latter quote, many respondents viewed discussions around death and dying as a *“taboo subject” [R3285]*, increasing the avoidance of having such conversations.

Finally, the fifth reason was that several respondents *“had not made any decision yet” [R2478]*. One respondent wanted to ensure they had reviewed all available information before deciding and having a well-informed discussion with them.

### Misconceptions

A further subset analysis of responses coded as misconceptions was reviewed at the request of NHSBT, with interest in whether these occurred from healthcare staff working with donors and recipients. Misconceptions were identified across the three questions, with misconceptions accounting for 24% of the responses to the against the legislation question. Responses used emotive, powerful words with suggestions of State ownership of organs, abuse of the system to procure organs, change in treatment of donors to hasten death, religious and cultural objections, and recipient worthiness.

*I worked in organ retrieval theatre during my career and I was uncomfortable with the way the operations were performed during this period. Although the 'brain dead' tests had been completed prior to the operation the vital signs of the patient often reflected that the patient was responding to painful stimuli. Sometimes the patient was not given the usual analgesia that is often given during routine operations. This made me rethink organ donation therefore I am uncomfortable with this. I always carried a donation card prior to my experience but subsequently would not wish to donate. This may be a personal feeling but that is my experience. [R660].*

*I think that this is a choice that should be left to individuals and families to make. After many years in nursing lots of it spent with transplant patients not all recipients embrace a 'healthy lifestyle' post-transplant with many going back to old lifestyle choices which made a transplant necessary in the first place. [R867].*

Additional comments suggested certain medical conditions and advancing age precludes donation and that the ability to choose which organs to donate had been removed.

*Most of them will be of no use as I have had a heart attack, I smoke and have Type 2 diabetes. [R595]*

Further analysis indicated that 27% (*n* = 24) of these comments were made by individuals who worked with or in an area that supported donors and recipients.

## Discussion

In summary, this qualitative paper has evidenced that the ability to make an autonomous informed decision is foremost in the respondent’s thoughts regarding an opt-out system. This has been commonly cited as a reason throughout the literature by those against an opt-out system [9, 10, 25, 26]. The loss of that ability was the primary reason for respondents being against the change in legislation with the notion that the decision is a personal choice cited as a reason for lack of discussion with family members. Respondents stated that the ability to make autonomous decisions needs to be adequately supported by evidence-based information that is accessible to all. If the latter is unavailable, they expressed concern for negative consequences. This includes an increase in the perceived belief of the potential for mistakes and abuse of the system, as well as family distress and loss of trust in the donation system and the staff who work in it, as supported by previous literature [9, 11].

Our findings further coincide with that of previous literature, highlighting views suggesting that the opt-out system is a move towards an authoritarian system, illustrating the commercialisation of organs, and a system that is open to abuse and mistakes [10–12, 27–29]. Healthcare staff require reassurance that the population, specifically the hard-to-reach groups like the elderly and homeless, have access to information and systems in order to be able to make an informed decision [30, 31]. Whilst the findings from the overall #options survey demonstrated awareness is higher in NHS staff, there was a significant narrative in the free-text response regarding a lack of awareness and a concern the general public must also lack the same awareness of the system change. Some responses also reflected medical mistrust concerns of the general public [13, 14, 16] as well as expressing a fear of losing trust with the public [9, 11, 16], as found within previous work. Additional research articles raising awareness of the opt-out system in England suggest that despite publicising the change with carefully crafted positive messaging, negative views and attitudes are likely to influence interpretation leading to an increase in misinformation [28]. Targeted, evidence-based interventions and campaigns that address misinformation, particularly in sub-groups like ethnic minorities, is likely to provide reassurance to NHS staff and the general public, as well as providing reliable resources of information [28].

Respondents also requested more detailed information about the process of organ donation. The disparity of information and the knowledge of the processes of donation includes eligibility criteria, perceived religious and cultural exclusions, practical processes of brain and circulatory death, and subsequent organ retrieval. As well as, most importantly, more information on the care provided to the donor before and after the donation procedure. The gap of available factual knowledge is instead filled by misconceptions and misunderstandings which is perpetuated until new information and knowledge is acquired. It may also be attributed to the increased awareness of ethical and regulatory processes. These attitudes and views illustrate the complexity of opinions associated with religion, culture, medical mistrust, and ignorance of the donation processes [11, 15, 32]. There is evidently a need for healthcare staff to display openness and transparency about the processes of organ donation and how this is completed, particularly with the donor’s family. It further reinforces the need to increase the knowledge of differing religious and cultural beliefs to support conversations with families [18, 19].

Both healthcare staff and the public would benefit from educational materials and interventions to address attitudes towards organ donation [19, 28, 33]. This would assist in correcting misconceptions and misunderstandings held by NHS staff, specifically those who support and work with organ donors and recipients. Previous work illustrates support for donation being higher in intensivists, recommending educational programmes to increase awareness across all healthcare staff [34]. The quantitative and qualitative findings of the #options survey would support this recommendation, adding that interventions need to be delivered by those working within organ donation and transplantation. This would build on the community work being conducted by NHSBT, hopefully leading NHS staff to become transplant ambassadors within their local communities.

A further finding was that of confusion and misunderstanding surrounding the role of the family, a finding also supported by the literature [11]. It was suggested that family distress would be heightened, and families would override the premise of opt-out. Literature also supports this could be further impacted if the family holds negative attitudes towards organ donation [20]. The uncertainty of the donors’ wishes was the most common reason for refusing from ethnic minority groups [35], further highlighting the need for family discussions. Without this, families feel they are left with no prior indication so they opt-out as a precaution. Making an opt-in decision known can aid the grieving process as the family takes comfort in knowing they are fulfilling the donors wishes [26] and reduces the likelihood of refusal due to uncertainty about their wishes [36]. The ambiguity around the role of the family, coupled with not explicitly stating a choice via the organ donor register or discussions with family can make it problematic for next of kin and NHS staff.

### Limitations

It is acknowledged that the findings of this study could have been influenced by the COVID-19 pandemic beyond the changes to the research delivery plan including a shift in critical care priorities, initial increase of false information circulating social media, delayed specialist nurse training, and removal of planned public campaigns [37, 38]. The degree of the impact is unknown and supports the view that ongoing research into healthcare staff attitudes is required. Additionally, the survey did not collect job titles and is therefore limited to combining all healthcare staff responses. It is understood not all staff, such as those working in mental health, would know in depth details of organ donation and legislation, but it is expected that their level of knowledge would be greater than that of the general public.

## Conclusions

The quantitative analysis [21] of the #options survey showed that overall NHS staff are well informed and more supportive of the change in legislation when compared to the general public. This qualitative analysis of the free-text responses provides a greater insight into the views of the healthcare staff who against the change. The reasons given reflect the known misconceptions and misunderstandings held by the general public and evidenced within the literature [9–16]. There are further concerns about the rationale for the change, the nature of the informed decision making, ease of access to information including information regarding organ donation processes. We therefore propose that educational materials and interventions for NHS staff are developed to address the concepts of autonomy and consent, are transparent about organ donation processes, and address the need for conversations with family. Regarding the wider public awareness campaigns, there is a continued need to promote the positives and refute the negatives to fill the knowledge gap with evidence-based information [39] and reduce misconceptions and misunderstandings.

### Supplementary Information

**Supplementary Material 1.**

**Supplementary Material 2.**

## Data Availability

The datasets analysed during the current study are available from the corresponding author on reasonable request.

## Abbreviations

COVID-19: Coronavirus Disease 2019
IRAS: Integrated research application system
NENC: North-East and North Cumbria
NHS: National Health Service
NHSBT: National Health Service Blood and Transplant
NIHR: National Institute of Health Research
ODR: Organ donor register
UK: United Kingdom

## References

[1] NHS Blood and Transplant. Max and Keira’s Law come into effect in England, https://www.organdonation.nhs.uk/get-involved/news/max-and-keira-s-law-comes-into-effect-in-england (Accessed 24 Feb 2021).

[2] NHS Blood and Transplant. Organ donation laws. https://www.organdonation.nhs.uk/uk-laws/ (Accessed 26 July 2023).

[3] Human Tissue Authority. Human Tissue Act 2004: human tissue authority. 2017. https://www.hta.gov.uk/policies/human-tissue-act-2004 (Accessed 07 Apr 2023).

[4] NHS Blood and Transplant. ODT clinical, donation after brainstem death. 2019. https://www.odt.nhs.uk/deceased-donation/best-practice-guidance/donation-after-brainstem-death/ (Accessed 07 Apr 2023).

[5] NHS Blood and Transplant. Organ and Tissue Donation and Transplantation: Activity Report 2022/23. https://nhsbtdbe.blob.core.windows.net/umbraco-assets-corp/30198/activity-report-2022-2023-final.pdf (Accessed 26 July 2023).

[6] NHS Blood and Transplant. Welsh Health Minister celebrates that ‘Opt-out organ donation scheme has transformed lives’. 2020. https://www.organdonation.nhs.uk/get-involved/news/welsh-health-minister-celebrates-that-opt-out-organ-donation-scheme-has-transformed-lives/#:~:text=Consent%20rates%20for%20donation%20have,from%20180%20in%202017%2F18 (Accessed 26 July 2023).

[7] Noyes J, McLaughlin L, Morgan K (2019). Short-term impact of introducing a soft opt-out organ donation system in Wales: before and after study. BMJ Open.

[8] Madden S, Collett D, Walton P, Empson K, Forsythe J, Ingham A, Morgan K, Murphy P, Neuberger J, Gardiner D (2020). The effect on consent rates for deceased organ donation in Wales after the introduction of an opt-out system. Anaesthesia.

[9] Rieu R (2010). The potential impact of an opt-out system for organ donation in the UK. Law, ethics and medicine.

[10] Miller J, Currie S, McGregor LM, O’Carroll RE. ‘It’s like being conscripted, one volunteer is better than 10 pressed men’: A qualitative study into the views of people who plan to opt-out of organ donation. 2020; 25: 257–274. 10.1111/bjhp.12406 10.1111/bjhp.12406 PMC7216962 31999878

[11] Miller J, Currie S, O’Carroll RE (2019). ‘If I donate my organs it’s a gift, if you take them it’s theft’: a qualitative study of planned donor decisions under opt-out legislation. BMC Public Health.

[12] Rudge CJ. Organ donation: opting in or opting out? British Journal of General Practice. 2018: 62–63. 10.3399/bjgp18X694445 10.3399/bjgp18X694445 PMC5774938 29371294

[13] Morgan SE, Stephenson MT, Harrison TR, Afifi WA, Long SD (2008). Facts versus ‘feelings’: how rational is the decision to become an organ donor?. J Health Psychol.

[14] Clark NL, Copping L, Swainston K, McGeechan GJ. Attitudes to Organ Donor Registration in England Under Opt-Out Legislation. Progress In Transplantation. 2023; 0(0). doi:10.1177/15269248231189869 10.1177/15269248231189869 37475461

[15] Miller J, Currie S, O’Carroll RE (2019). ‘What if I’m not dead?’ – Myth-busting and organ donation. Br J Health Psychol.

[16] Organ Donation Taskforce. The potential impact of an opt out system for organ donation in the UK. The National Archives. 2008:1–36.

[17] Morgan M, Kenten C, Deedat S, Farsides B, Newton T, Randhawa G (2016). Increasing the acceptability and rates of organ donation among ethnic groups: a programme of observational and evaluative research on Donation, Transplantation and Ethnicity (DonaTE). Program Grants Appl Res.

[18] Doerry K, Oh J, Vincent D, Fishcer L, Schulz-Jurgensen S (2022). Religious and cultural aspects of organ donation: Narrowing the gap through understanding different religious beliefs. Pediatr Transplant.

[19] Witjes M, Jansen NE, van der Hoeven JG, Abdo WF (2019). Interventions aimed at healthcare professionals to increase the number of organ donors: a systematic review. Crit Care.

[20] Shepherd L, O’Carroll RE, Ferguson E (2023). Assessing the factors that influence the donation of a deceased family member’s organs in an opt-out system for organ donation. Soc Sci Med.

[21] Coe D, Newell N, Jones M, Robb M, Clark N, Reaich D, Wroe C (2023). NHS staff awareness, attitudes and actions towards the change in organ donation law in England – results of the #options survey 2020. Archives of Public Health.

[22] Welsh Government Social Research. Survey of Public Attitudes to Organ Donation: Wave 2. 66/2013, Welsh Government, 2013. https://gov.wales/sites/default/files/statistics-and-research/2019-04/public-attitudes-organ-donation-wave-2.pdf (Accessed 29 June 2021).

[23] Tong A, Sainsbury P, Craig J (2007). Consolidated criteria for reporting qualitative research (COREQ): a 32-item checklist for interviews and focus groups. Int J Qual Health Care.

[24] Braun V, Clarke V (2006). Using thematic analysis in psychology. Qual Res Psychol.

[25] Cantrell TK (2019). The ‘opt-out’ approach to deceased organ donation in England: A misconceived policy which may precipitate moral harm. Clin Ethics.

[26] Hoeyer K, Jensen AMB, Olejaz M (2015). Transplantation as an abstract good: practising deliberate ignorance in deceased organ donation in Denmark. Sociol Health Illn.

[27] Dalal AR (2015). Philosophy of organ donation: Review of ethical facets. World J Transplant.

[28] Faherty G, Williams L, Noyes J, McLaughlin L, Bostock J, Mays N (2022). Analysis of content and online public responses to media articles that raise awareness of the opt-out system of consent to organ donation in England. Front Public Health.

[29] Koplin JJ (2015). From blood donation to kidney sales: the gift relationship and transplant commercialism. Monash Bioeth Rev.

[30] Morgan M, Kenten C, Deedat S (2013). Attitudes to deceased organ donation and registration as a donor among minority ethnic groups in North America and the UK: a synthesis of quantitative and qualitative research. Ethn Health.

[31] Truijens D, van Exel J (2019). Views on deceased organ donation in the Netherlands: A q-methodology study. PLoS ONE.

[32] Irving  MJ, Tong  A, Jan  S, Cass  A, Rose  J, Chadban  S, Allen  RD, Craig  JC, Wong G, Howard K (2012). Factors that influence the decision to be an organ donor: a systematic review of the qualitative literature. Nephrol Dial Transplant.

[33] Radunz S, Hertel S, Schmid KW, Heuer M, Stommel P, Fruhauf NR, Saner FH, Paul A, Kaiser GM (2010). Attitude of Health Care Professionals to Organ Donation: Two Surveys Among the Staff of a German University Hospital. Transplant Proc.

[34] Umana E, Grant O, Curran E, May P, Mohamed A, O’Donnell J (2018). Attitudes and knowledge of healthcare professionals regarding organ donation. A survey of the Saolta University health care group. Ir Med J.

[35] Daga S, Patel R, Howard D, et al. ‘Pass it on’ - New Organ Donation Law in England May 2020: What Black, Asian or Minority Ethnic (BAME) Communities should do and Why? The Physician; 6. Epub ahead of print 5 May 2020. DOI: 10.38192/1.6.1.7.

[36] Truijens D, van Exel J (2019). Views on deceased organ donation in the Netherlands: A q-methodology study. PLoS ONE.

[37] NHS Organ Donation. The UK Opt-Out Experience. https://www.youtube.com/watch?v=22oCq5NKoiE&t=3211s. YouTube 25 February 2021. Accessed 05 Dec 2023.

[38] UK Parliament, House of Lords, Lords Chamber, Volume 803: debated 18 May 2020. Draft Human Tissue (Permitted Material: Exceptions) (England) Regulations 2020. https://hansard.parliament.uk/lords/2020-05-18/debates/1a7747af-1951-4289-b8d5-639493c85bb1/LordsChamber. 18 May 2020. Accessed 05 Dec 2023.

[39] Miller J, McGregor L, Currie S, O’Carroll RE (2022). Investigating the effects of threatening language, message framing, and reactance in opt-out organ donation campaigns. Ann Behav Med.

